# Functions of autophagy in plant carbon and nitrogen metabolism

**DOI:** 10.3389/fpls.2014.00301

**Published:** 2014-06-24

**Authors:** Chenxia Ren, Jingfang Liu, Qingqiu Gong

**Affiliations:** Tianjin Key Laboratory of Protein Science and Department of Plant Biology and Ecology, College of Life Sciences, Nankai UniversityTianjin, China

**Keywords:** autophagy, carbon, nitrogen, chloroplast, starch

## Abstract

Carbon and nitrogen are essential components for plant growth. Although models of plant carbon and nitrogen metabolisms have long been established, certain gaps remain unfilled, such as how plants are able to maintain a flexible nocturnal starch turnover capacity over various light cycles, or how nitrogen remobilization is achieved during the reproductive growth stage. Recent advances in plant autophagy have shed light on such questions. Not only does autophagy contribute to starch degradation at night, but it participates in the degradation of chloroplast proteins and even chloroplasts after prolonged carbon starvation, thus help maintain the free amino acid pool and provide substrate for respiration. The induction of autophagy under these conditions may involve transcriptional regulation. Large-scale transcriptome analyses revealed that *ATG8e* belongs to a core carbon signaling response shared by *Arabidopsis* accessions, and the transcription of *Arabidopsis ATG7* is tightly co-regulated with genes functioning in chlorophyll degradation and leaf senescence. In the reproductive phase, autophagy is essential for bulk degradation of leaf proteins, thus contributes to nitrogen use efficiency (NUE) both under normal and low-nitrogen conditions.

## INTRODUCTION

Eukaryotic cells carry out autophagy to clean up the house and keep fit (Yang and Klionsky, 2010). The hallmark of autophagy is the formation of a double-membrane vesicle, the autophagosome, and its subsequent fusion with the lysosome or the lytic vacuole (Mizushima et al., 2011). The cargoes inside the autophagosome are then degraded; free amino acids are released back into the cytosol (Mizushima et al., 2011). Conserved from yeasts to plants, this bulk degradation pathway is highly efficient in turning over proteins and organelles, and has an essential role in maintaining free amino acid pools upon starvation (Onodera and Ohsumi, 2005; Thompson and Vierstra, 2005). Defects in autophagy compromises plant vitality and disease resistance mostly in a salicylic acid signaling-dependent way (Liu et al., 2005; Yoshimoto et al., 2009; Lai et al., 2011; Lenz et al., 2011; Wang et al., 2011). Autophagy mutants are generally sensitive towards abiotic stresses (Liu et al., 2009; Zhou et al., 2013), have lower levels of anthocyanin biosynthesis (Masclaux-Daubresse et al., 2014), and produce less seeds than the wild-type (Hanaoka et al., 2002; Guiboileau et al., 2012).

Studies over the past 15 years have successfully defined the autophagy process in plants (Liu and Bassham, 2012; Li and Vierstra, 2012). Nearly all core machinery AuTophaGy (ATG) proteins identified based on their sequence homology to the yeast and mammalian homologs (Xie and Klionsky, 2007). Molecular functions of the plant ATGs have been verified both through *in vivo*, genetic and physiological studies (Liu and Bassham, 2012; Li and Vierstra, 2012) and *in vitro* reconstitution (Fujioka et al., 2008). The basic mechanisms of plant autophagy now have been confirmed to be similar to those of yeasts and animals.

Moreover, plant-specific, autophagy-related genes and functions have been discovered (Ishida et al., 2008; Wada et al., 2009; Izumi et al., 2010, 2013; Honig et al., 2012; Ono et al., 2013; Wang et al., 2013). Through these findings, a unique link between autophagy and plant carbon status can be seen. Also different from the yeast, plant autophagy genes are regulated not only post-transcriptionally (Suttangkakul et al., 2011; Li et al., 2014), but transcriptionally. Recent studies have also revealed a function for autophagy in nitrogen remobilization (Guiboileau et al., 2012, 2013; Xia et al., 2012), thus pointing out a new direction for the study of plant nitrogen metabolism and yield formation. More details are discussed hereafter.

## TRANSCRIPTION OF PLANT *ATG* GENES ARE REGULATED BY CARBON AND NITROGEN STATUS

Most yeast *ATG* genes are not regulated transcriptionally. For instance, upon nitrogen starvation, only *ATG8* and *ATG14* are promptly and significantly induced (Kirisako et al., 1999; Chan et al., 2001). In contrast, many plant *ATG* genes are transcriptionally regulated. The mRNA levels of rice *ATG* genes have been reported to be strongly regulated by nitrogen level, abiotic stresses, and hormones (Xia et al., 2011). Sucrose starvation induced waves of expression of core machinery *ATG* genes in *Arabidopsis* suspension culture (Rose et al., 2006). In tobacco leaves, transcript levels of several *ATG* genes are elevated during the night (Wang et al., 2013). Furthermore, transcription of individual *ATG8* and *ATG18* genes is regulated differently upon carbon and nitrogen starvation, and further exhibits tissue-specificity (Yoshimoto et al., 2004; Xiong et al., 2005; Xia et al., 2012).

**FIGURE 1:**
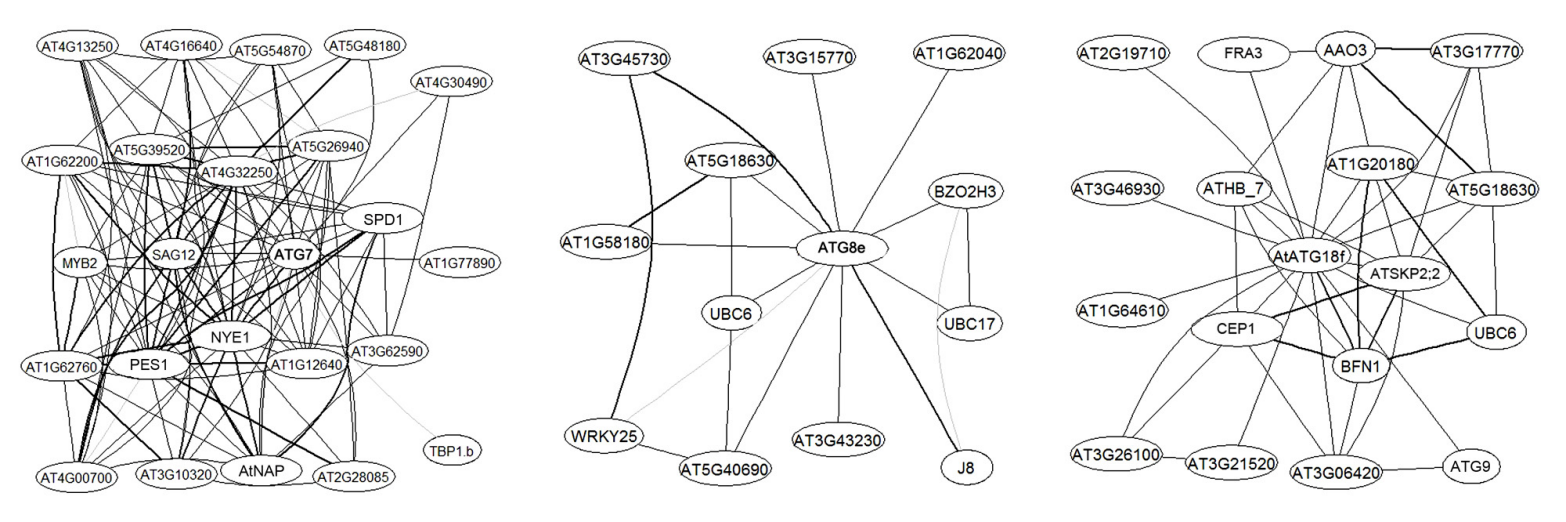
***AtATG7*, *AtATG8e*, and *ATG18f* are hubs in *Arabidopsis* transcriptional networks.** The network was built as described in Ma et al. (2007). Sub-networks centered on the *ATG* genes with a maximum of two steps are extracted from the expanded network. Genes are labeled with their primary gene symbols (TAIR10) wherever possible. AGI numbers are provided otherwise. Edges, i.e., links between nodes, represent co-expression. Correlation levels are represented by the width of the lines, with the boldest lines indicating top 20% correlation values.

More importantly, large-scale analyses have suggested the possible involvement of certain *ATG* genes in plant carbon metabolism and signaling. *ATG8e* was identified as one of 26 genes that constitute a robust core of a carbon signaling response shared by a large number of *Arabidopsis* accessions (Sulpice et al., 2009). In a graphical Gaussian model (GGM) constructed over 2000 *Arabidopsis* Affymetrix gene chips which captures only very strong correlations in transcript levels (Ma et al., 2007), several *ATG* genes emerged as hubs of sub-networks (**Figure 1**). For instance, *ATG7*, encoding the E1-like activating enzyme for both ATG8 and ATG12 conjugation, is surrounded by key regulators and marker genes of leaf senescence such as *MYB2*, *AtNAP*, *SAG12*, and *NYE1* (**Figure 1**). According to the guilty by association rule, *ATG7* is likely a hub during plant senescence, when carbon is used for leaf energy and nitrogen gets remobilized (Diaz et al., 2008). Clearly, compared with unicellular eukaryotes, higher plants have extended the regulatory repertoire to better adapt to the changing environment and to efficiently allocate essential resources throughout their lifespan.

## AUTOPHAGY PARTICIPATES IN STARCH BREAKDOWN

The diurnal cycle has a great impact on the life of a plant. During the day, the plant fixes carbon; at night, remobilization of starch supports respiration and growth. An intriguing fact about starch break down is that the rate can be adjusted to suit a range of day lengths, always with little left by dawn (Smith and Stitt, 2007), thus enabling the plant to maintain a maximum growth rate possible. Genetic and biochemical studies have established the starch degradation pathway (Stitt and Zeeman, 2012), and regulation of starch degradation has been shown to be circadian rhythm-dependent (Graf et al., 2010). Nevertheless, new questions have been raised, such as what exactly the clock signals are and how they are integrated with the information on the remaining amount of starch (Stitt and Zeeman, 2012).

The newly reported, autophagy-dependent starch degradation pathway has shed some light on the questions (Wang et al., 2013). Several core machinery *ATG* genes are transcriptionally regulated by the diurnal cycle (Wang et al., 2013). The number of autophagosomes gets higher before dusk, and goes back to normal by dawn. In contrast to the wild-type, several *atg* mutants have starch left on their plates in the morning (Wang et al., 2013). Interestingly, the starch granules that are transported into the vacuole by autophagosomes are much smaller than the remaining ones in the chloroplast, suggesting that the autophagy-dependent pathway might be a complement to the classic degradation pathway (Wang et al., 2013).

## AUTOPHAGY IS INDUCED BY LEAF CARBON DEFICIENCY TO MAINTAIN ENERGY LEVELS

The chloroplast is not only the site for photosynthesis, but stocks 75–80% of total leaf nitrogen (Makino and Osmond, 1991). Transcriptome analyses showed that, when a plant is severely challenged by stresses, suppression of chloroplast activities and activation of protein turnover pathways (including autophagy) both happen at the same time (Gong et al., 2005; Ma et al., 2006). During leaf senescence, not only proteins inside the chloroplast but also pieces of chloroplast are recycled (Hortensteiner and Feller, 2002; Otegui et al., 2005; Martinez et al., 2008a). Whether such degradation involves autophagy has unsurprisingly become a hot topic in recent years.

Anyone who has worked with protoplasts may have noticed that, after kept in the dark for a prolonged period of time, chloroplasts within a single mesophyll protoplast become less in number and smaller in size (Contento et al., 2005). Although chloroplast protein turnover have been studied extensively (Hortensteiner, 2006; Martinez et al., 2008b), recently identified autophagy-dependent chloroplast protein degradation further advanced our understanding of the process, as reviewed recently (Ishida et al., 2014). After dark treatment (combined with vacuolar H^+^-ATPase inhibitor Concanamycin A), *Arabidopsis* mesophyll cells accumulate RuBisCO-containing bodies (RCBs) and structures containing pieces of chloroplasts in the lytic vacuole, whereas in* atg4a atg4b-1* double mutants neither can be seen (Ishida et al., 2008; Wada et al., 2009). Consistently, the number of chloroplasts is not reduced in *atg4a atg4b-1* mesophyll cells after prolonged carbon starvation, and the size of chloroplasts is only partially reduced (Wada et al., 2009). RCBs also appeared to be more sensitive to carbon starvation than to nitrogen starvation, and by adding carbohydrates to the culture, accumulation of RCBs is inhibited (Izumi et al., 2010). Futhermore, starchless mutants *pgm1-1* and *adg1-1* accumulate more RCBs than the wild-type, whereas less RCBs can be seen in starch-excess mutants *sex1-1* and *mex1-3*, suggesting that this specific form of plant autophagy may be controlled by starch levels (Izumi et al., 2010). Finally, in the latest report by Izumi et al. (2013) autophagy was suggested to contribute to the maintenance of the free amino acid pool during carbon starvation, thus providing energy source for respiration.

## AUTOPHAGY CONTRIBUTES TO NITROGEN REMOBILIZATION AND SEED PRODUCTION

Nitrogen is an essential element for plants. To turn soil nitrogen into macromolecules such as amino acids, nucleic acids, and chlorophyll, nitrogen uptake, assimilation, translocation, and remobilization must be coordinately executed by the plant (Masclaux-Daubresse et al., 2010; Xu et al., 2012; Avila-Ospina et al., 2014). Recent studies have illuminated the functions of plant autophagy in nitrogen remobilization both under starvation conditions and during normal growth phases.

Nitrogen starvation has been used by yeast, animal, and plant researchers as a standard procedure to induce autophagy. The *Arabidopsis* autophagy mutants, such as *atg5*, *atg10*, *atg13a atg13b*, and *ATG18a RNAi*, are all less tolerant to nitrogen limitation compared to the wild-type (Thompson et al., 2005; Xiong et al., 2005; Phillips et al., 2008; Suttangkakul et al., 2011), confirming a role for autophagy in nitrogen recycling. Consistently, over-expression of *GmATG8c*, an *ATG8* homolog from soybean, confers tolerance towards nitrogen limitation both in soybean calli and in transgenic *Arabidopsis* (Xia et al., 2012).

After a transition from vegetative phase into reproductive phase, a plant produces seeds to complete its life cycle. At this stage, leaves generally have started to senesce, and nitrogen source obtained from uptake and assimilation is usually not enough to support seed development (Lim et al., 2007). Leaf nitrogen remobilization thus becomes a critical step during seed maturation (Masclaux-Daubresse et al., 2010).

Guiboileau et al. (2012) discovered that, in several *atg* mutants and RNAi plants, nitrogen use efficiency (NUE), represented by the nitrogen harvest index (NHI): Harvest index (HI) ratio, is lower than that of the wild-type both at the nitrogen-rich condition (+N) and over nitrogen limitation (-N). The lower NUE was shown to be independent of seed productivity (Guiboileau et al., 2012). They also demonstrated that the lower NUE of *atg* mutants is due to a defect of nitrogen remobilization leading to the accumulation of undigested soluble proteins in their leaves (Guiboileau et al., 2013). Similarly, using transgenic *Arabidopsis* lines carrying *35S:GmATG8c*, we found that the transgenic lines with higher levels of autophagy have comparable nitrogen concentrations to the wild-type at –N condition, yet maintain a higher biomass at both +N and –N conditions, and enter the reproductive phase earlier to produce more branches and more siliques at +N condition (Xia et al., 2012). Upon seed maturation, the transgenic lines also had slightly but significantly more seeds in each silique, however, the 1000 grain weight stays unchanged (Xia et al., 2012). These results indicate that a higher level of autophagy can better facilitate the flux of nitrogen from source to sink, thus enabling more flower production and subsequent seed setting. Taken together, autophagy can be considered as an essential factor in nitrogen remobilization.

## PERSPECTIVES

So far, studies have elucidated many basic molecular mechanisms and physiological and pathological consequences of autophagy in plants. The relationships between autophagy and plant carbon and nitrogen metabolism have started to be revealed. It can be expected that in the coming years, more interesting and fundamental researches will emerge to solve more existing problems in plant cell biology and plant metabolism. For instance, is there a common set of transcription regulators for the induction of plant *ATG* genes? Construction of higher-order gene regulatory networks will certainly be useful. Can the newly identified role of autophagy in starch degradation be integrated into the classic model of nocturnal starch turnover? The core machinery genes are generally controlled by the circadian rhythm; however, are they directly linked to the yet unidentified clock signals? Both mathematical modeling and well-planned screening may help answer these questions. Finally, the interaction between carbon and nitrogen has always been a vital topic in plant metabolism and signaling, and autophagy now appears to have a leading role (Guiboileau et al., 2013). The detailed molecular mechanism behind the link still waits to be explored. Given the importance of autophagy in maintaining cell homeostasis and plant vitality, future discoveries will not only advance our understanding in plant autophagy, but also surely be applicable in crop improvement.

## Conflict of Interest Statement

The authors declare that the research was conducted in the absence of any commercial or financial relationships that could be construed as a potential conflict of interest.
